# Supplementary material for: Fracture Mechanics and Oxygen Gas Barrier Properties of Al2O3/ZnO Nanolaminates on PET Deposited by Atomic Layer Deposition
Source: Nanomaterials (Basel). 2019 Jan 11;9(1):88. doi: 10.3390/nano9010088 (PMC6359614; doi:10.3390/nano9010088)
Supplement: Supplementary file 1 [file nanomaterials-09-00088-s001.pdf]

Supplementary Information

1. ALD Cycle Parameters

The total number of cycles was fixed at 1000 cycles for all the depositions. Single ZnO and Al_2O_3 coatings were also deposited by using 1000 cycles and characterized as reference samples. The total film thicknesses of the Al_2O_3/ZnO nanolaminates were measured on fragments which delaminated from the PET substrate as a result of the tensile strain experiment. The bilayer thickness (one stack of Al_2O_3 and ZnO) was calculated by dividing the total film thickness by the number of bilayers.

Table S1. ALD deposition sequences of investigated Al_2O_3/ZnO nanolaminates, total film thickness on PET as measured by SEM and resulting bilayer thicknesses.

Samples	Number of Al_2O_3 Cycles	Number of ZnO Cycles	Number of Bilayers	Total Thickness (nm)	Nominal Bilayer Thickness (nm)
ZnO	0	1000	0	210 ± 38	single
Al_2O_3	1000	0	0	200 ± 19	single
2 × 130 nm	250	250	2	260 ± 22	130
10 × 25 nm	50	50	10	250 ± 26	25
50 × 4.8 nm	10	10	50	240 ± 29	4.8

2. SEM Thickness Observations

SEM cross-section observations were performed using an emission field Hitachi S-4800 microscope on the films deposited on PET, for samples that had undergone the tensile testing. Light contrast comes from the ZnO material. ZnO films were measured as is, the Al_2O_3 sample prepared by sputtering a thin (<10 nm) gold layer on top, and the nanolaminate samples were transferred to carbon tabs by pressing the sample against the conductive adhesive allowing the delaminated film to be electrically grounded to avoid charging during SEM observation.

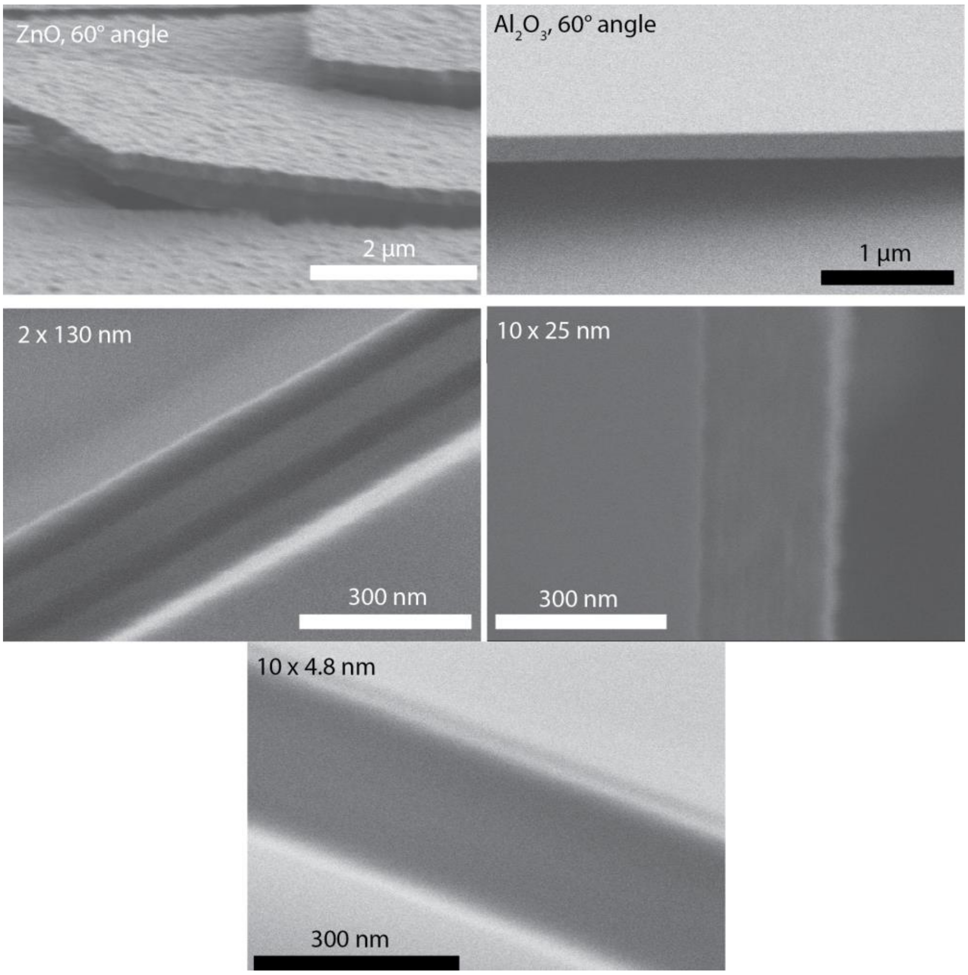

Figure S1. SEM cross-sections of coating fragments of the various nanolaminates after tensile experiments. Light contrast comes from the ZnO material single.

3. STEM Cross-Sections and Failure Analysis

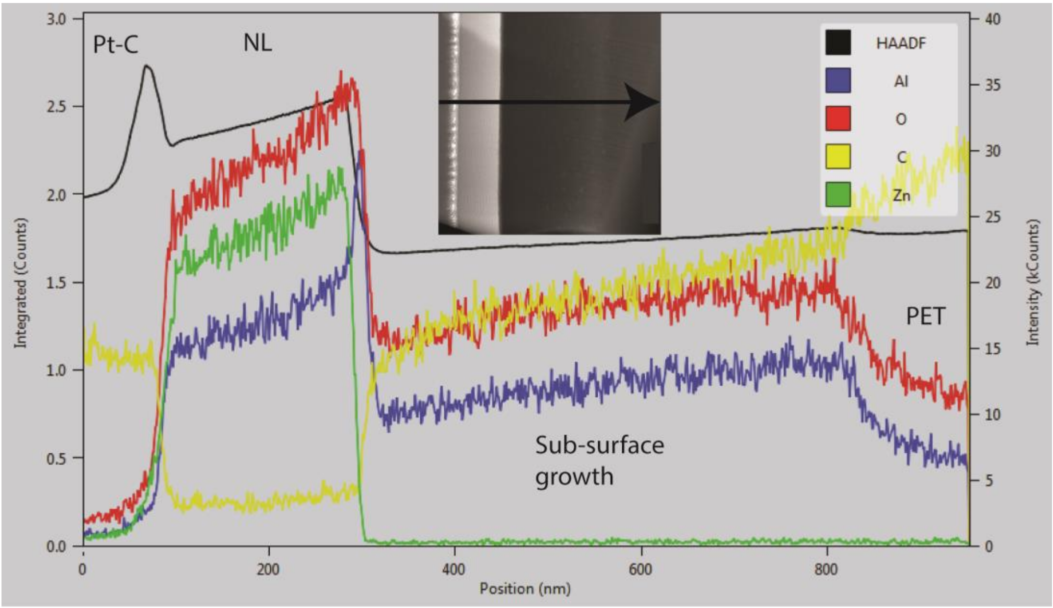

Figure S2. EDS line scan of 50 × 4.8 nm sample and sub-surface growth, shown in Figure 4b. The sub-surface growth contains Al, O, and C.

4. Sample Data Obtained from Tensile Experiments

Table S2. Tensile experiment results for individual samples. The empty values represent ones that could not be accurately determined from the measurement data or Weibull fit. Uncertainties are listed after each value.

**ZnO/Sample Acronym**	**COS (%)**	**CD (mm^{-1})**	**Cohesive Strain (%) Film**	**± (%)**	**Cohesive Strength (MPa) Film**	**± (MPa)**	**Interfacial Shear Stress (MPa)**	**± (MPa)**
S1	0.817	143.5						
S3	0.846	152.9	1.06	0.16	1540.67	247.30	66.14	9.67
S5		137.2	0.71	0.82	1024.78	1193.01	39.48	7.38
S6		146.6	1.07	0.61	1552.96	905.84	63.92	10.87
S7	0.709	134.2	1.17	0.09	1690.54	150.29	63.70	9.25
S4	0.689	137.2	1.04	0.45	1505.47	663.36	57.99	9.19

**Al_2O_3**	**COS (%)**	**CD (mm^{-1})**	**Cohesive Strain (%) Film**	**± (%)**	**Cohesive Strength (MPa) Film**	**± (MPa)**	**Interfacial Shear Stress (MPa)**	**± (MPa)**
S1	0.514							
S3	0.499	40	1.18	0.57	1935.03	955.71	20.70	3.55
S5	0.486	41.6	0.68	0.39	1107.57	647.72	12.32	1.92
S6	0.447	38.285	0.76	0.38	1239.89	637.26	12.69	1.98
S7		34.95	0.95	0.25	1561.75	417.54	14.60	2.17

**2 × 130 nm**	**COS (%)**	**CD (mm^{-1})**	**Cohesive Strain (%) Film**	**± (%)**	**Cohesive Strength (MPa) Film**	**± (MPa)**	**Interfacial Shear Stress (MPa)**	**± (MPa)**
S2	0.225	51.76	0.51	0.37	770.52	570.02	13.86	2.12
S3	0.837	70.5	1.13	0.80	1714.88	1231.86	42.03	7.89
S7	0.431	43.36						
S8	0.502	51.76	0.55	0.36	827.99	550.65	14.90	2.27
S9	0.366	49.56	0.63	0.43	955.24	662.10	16.46	2.58

**50 × 4.8 nm**	**COS (%)**	**CD (mm^{-1})**	**Cohesive Strain (%) Film**	**± (%)**	**Cohesive Strength (MPa) Film**	**± (MPa)**	**Interfacial Shear Stress (MPa)**	**± (MPa)**
S1	0.607							
S3	0.594	44.70	1.50	1.20	2111.20	1710.63	30.28	6.71
S44	0.482	49.67	1.46	0.32	2057.19	466.13	32.79	6.70
S4	0.655	52.04						
S5	0.476	36.05	0.60	0.11	840.02	163.44	9.72	1.90

10 × 25 nm	COS (%)	CD (mm^{-1})	Cohesive Strain (%) Film	± (%)	Cohesive Strength (MPa) Film	± (MPa)	Interfacial Shear Stress (MPa)	± (MPa)
S2	0.722	33	1.64	1.90	2398.30	2791.59	25.55	36.77
S3	0.49	32	0.65	0.63	949.91	930.22	9.82	10.30
S4	0.615	39						
S5	0.62	41						
S6	0.563	43	0.69	0.46	1004.70	683.25	13.94	7.90
S7	0.398	33						
